# Huidouba Improved Podocyte Injury by Down-Regulating Nox4 Expression in Rats With Diabetic Nephropathy

**DOI:** 10.3389/fphar.2020.587995

**Published:** 2020-11-30

**Authors:** KunBao Yang, YingHui Bai, Ning Yu, BiNan Lu, GuiYan Han, ChangJiang Yin, ZongRan Pang

**Affiliations:** ^1^ School of Pharmacy, Minzu University of China, Beijing, China; ^2^ Hebei Key Laboratory of Research and Development for Chinese Medicine, Chengde Medical University, Hebei, China; ^3^ The Affiliated Hospital of Chengde Medical University, Hebei, China

**Keywords:** Huidouba, Nox4, podocyte, diabetic nephropathy, oxidative injury

## Abstract

Diabetic nephropathy (DN), as the most common microvascular complication of diabetes mellitus (DM), has become one of the leading causes of end-stage renal disease (ESRD). Numerous studies have indicated that podocyte loss plays an important role in the development of DN and can even cause proteinuria in the early stage of DN. In the study, we found that Huidouba (HDB) significantly decreased the level of fasting blood glucose (FBG), the ratio of microalbumin to urine creatine (mAlb/Ucr), serum creatine (Scr), serum urea nitrogen (BUN), and malondialdehyde (MDA) in the kidney and downregulated the expression of Nox4 predominantly located in glomerular tissue while upregulating nephrin and WT1 expression in DN rats. In addition, HDB could also reduce podocyte damage and glomerular basement membrane (GBM) pathologic changes, as shown by transmission electron microscopy (TEM). *In vitro* study showed that HDB could inhibit high glucose (HG)-induced Reactive Oxygen Species (ROS) production and protect against podocyte apoptosis by downregulated Nox4 expression in podocytes. These results may provide a scientific basis for developing HDB as a potential folk medicine for the treatment of DN.

## Introduction

At the end of 2017, the International Diabetes Federation (IDF) released the eighth edition map of the global epidemic of diabetes (DM) (Sanz et al., 2018). The map showed that the global number of patients with DM aged 20–79 years had reached 425 million. If this increase is not halted, there will be 642 million people with the disease by 2045. China has the largest population with DM, which amounts to 114.4 million (Unnikrishnan et al., 2017). DN, as the most common microvascular complication of DM, has become one of the leading causes of ESRD (Marathe et al., 2017). The latest statistics from Peking University Hospital showed that DN has become the leading cause of ESRD in China. There is a total of 24 million people with DN in China, and the prevalence of DN has already surpassed that of glomerulonephritis related to chronic kidney disease (Zhang et al., 2016). At present, the clinical treatment of DN mainly aims to control blood glucose and blood pressure, but it cannot effectively prevent the exacerbation of DN. Therefore, further study of the pathogenesis is needed to search for an effective therapy for DN. It is of great importance to delay the progression of DN.

Traditional Chinese medicine (TCM) maintains that DN is due to the lower wasting thirst of Xiaoke disease. Failure or mismanagement of Xiaoke would lead to Qi and Yin deficiency and spleen and kidney deficiency, which may finally result in Qi-Blood-Yin-Yang deficiency, kidney collateral stasis and turbid toxic retention. Yiqiyangyin is an effective treatment for early DN in the clinic (Zhang and Gao 2015). TCM and folk medicines have the advantages of reducing blood glucose, blood pressure and DM microvascular complications. Therefore, finding safe and effective diabetes medications in natural drugs has drawn increasing attention from scientists. HDB, as a Tibetan medicine, is also called “close pocket” and is derived from the nest of *Atypus karschi* Doenitz, which is found around the roots of old tea trees in the Mount Emei area of Sichuan Province. It has a unique effect on Yiqiyangyin and nourishes kidney yin, eases DM symptoms, promotes the natural balance of blood glucose, harmonizes Qi-Blood and prevents the progression of DN. As a folk prescription, HDB has been passed down from generation to generation for thousands of years in the Mount Emei area of Sichuan Province. It enjoys a reputation as a “wonder drug” of Mount Emei, an invincible opponent of DM, and a natural green treasure from Nature (Yang and Pang 2017). We found that HDB has an obvious therapeutic effect on DN. It could downregulate the expression of Nox4, which is predominantly localized in glomerular tissue and relieve oxidative injury in podocytes and proteinuria in DN rats.

## Materials

### Animals

Fifty five-week-old male Sprague-Dawley (SD) rats weighing 120–140 g were purchased from Beijing HFK Bioscience Co., Ltd. (Beijing, China) (animal certificate number: SCXK [JING]-2014-0004). The rats were kept in an ambient temperature (23 ± 1°C) and relative humidity (55 ± 5%) environment with 12 h light-dark cycles. There were five rats per cage, and all rats were allowed free access to water and food. All animal procedures were approved by the Laboratory Animal Ethics Committee of Chengde Medical University and complied with the Guidance for the Care and Use of Laboratory Animals.

### Cell Culture and Treatment

Conditionally immortalized mouse podocytes (MPC5) were obtained from ATCC (Manassas, VA). The recovered cells were cultured and subcultured for 5 days in RPMI-1640 medium containing 10% FBS, 1 μL/ml penicillin, 1 μL/ml streptomycin, and 20 U/ml interferon-γ at 33°C in a humidified incubator, 5% CO_2_, 95% air atmosphere. Proliferated podocytes were thermoshifted to 37°C in the absence of interferon-γ for 14 days. When cells were then well-differentiated, they were maintained in a serum-deprived condition (0.25% FBS) for 24 h. Drug-containing serum was prepared as follows. Healthy male SD rats were gavaged twice a day with metformin (MET, 45 mg/kg) or different doses of HDB (HDBL, 110 mg/kg; HDBM, 130 mg/kg; HDBH, 180 mg/kg) for 3 days. Blood was collected from the abdominal aorta 1 h after the last administration, and was then centrifuged at 10,000 r/min for 15 min at 4°C to obtain serum. The serum was inactivated at 56°C for 30 min, sterilized with microporous membrane of 0.22 μm thick, frozen into blocks, and vacuum-dried under −70°C. The podocytes were treated with normal glucose (NG, 5.5 mmol/L) or high glucose (HG, 30 mmol/L) for 48 h in the absence or presence of drug-containing serum aforementioned and were then collected for proposed experiments.

### Drugs

HDB was purchased from Double Town in the Mount Emei area in Sichuan Province. It was harvested in June and sun-cured. Based on the pilot experiments and original drug regimen (Yang and Pang 2017), we processed the HDB as follows. The crude drug was washed gently, steeped in water for 2 h before processing, and extracted three times in boiling water (100°C) for 2 h after adding 9 or 10 volumes of water. The decoction was condensed to the appropriate relative density (1.02) under reduced pressure (70°C), and the concentrate was cooled to room temperature. A volume of 80% ethanol was added, and the concentrate was allowed to settle for 12 h at 4°C, after which it was sifted through a 147-μm mesh. The precipitate was collected and finally dried by a vacuum at reduced pressure. We entrusted the Chengde Jingfukang Pharmaceutical Group (JFKPG) to prepare HDB aqueous extract according to the above procedure. Metformin (MET) in 98% 1,1-dimethyl diguanidine hydrochloride was kindly gifted by Shijiazhuang Polee Pharmaceutical Co., Ltd. Streptozotocin (STZ, S0130) was purchased from the Sigma Company.

### Reagents and Instruments

The creatinine assay kit (CRE, No. C011-2-1), blood urea nitrogen assay kit (BUN, No. C013-2-1), and urine microalbuminuria assay kit (U-mAlb, No. E038-1-1) were purchased from Nanjing Jiancheng Bioengineering Institute. The ROS Assay kit (No. S0033S), β-Actin mouse monoclonal antibody (No. AF0003) and a protease and phosphatase inhibitor cocktail for general use (50X) (No. P1045) were purchased from Beyotime Institute of Biotechnology. The Lipid Peroxidation (MDA) Assay kit (No. ab118970), H&L (HRP) IgG (No. ab97051), rabbit monoclonal [UOTR1B493] to NADPH oxidase 4 (No. ab133303), and rabbit polyclonal to nephrin (No. ab58968) were purchased from Abcam Trading (Shanghai) Co., Ltd. The goat anti-rabbit IgG DyLight 488 (No. A23220) was purchased from Abbkine Scientific Co., Ltd. The goat anti-mouse IgG (H&L) Cy3 (No. GB21301), and DAPI (No. G1012) were purchased from Wuhan Servicebio Technology Co., Ltd. The Thiazolyl Blue Tetrazolium Bromide (MTT, No. M2128) was purchased from the Sigma Company.

The following instruments were used in our study: a fully automatic biochemical analyzer (BK200, Boko, China), multiscan spectrum (VarioskanFlash 3001, Thermo Scientific, United States), automatic tissue hydroextractor (Thermo-Excelsior-ES, United States), tissue embedder (Histocenter 3, Thermo Scientific, United States), rotary microtome (RM2125, Leica, Germany), water bath and flattening table combination (TK-218, China), ultralow temperature freezer (MDF-U4086S, Sanyo, Japan), inverted fluorescence microscope (Eclipse Ti-SR, Nikon, Japan), digital fluorescence microscope (DS-Ri1-U3, Nikon, Japan), SDS-PAGE electrophoresis system (DYCZ-24DN, LIUYI, China), transfer box (DYCZ-40D, LIUYI, China), chemiluminescence imaging system (Tanon-5200, China), humidified incubator (371, Thermo Fisher Scientific, United States), flow cytometry (BD Biosciences, United States) and transmission electron microscope (HT7700, HITACHI, TAC, Japan).

## Methods

### Model Establishment and Grouping

We performed unilateral nephrectomy plus high fat diet (HFD) feeding and intraperitoneal injection (i.p.) of STZ (35 mg/kg) to induce the DN model. After 1 week of adaptive feeding, eight rats were randomly chosen for the sham operation control (SC) group. The other rats were anesthetized with an intraperitoneal injection of ketamine (10 mg/100 g BW) and then subjected to right nephrectomy (at 0 weeks), while rats in the SC group were only exposed at the right kidney during the operation. All rats were intraperitoneally injected with 100,000 units benzylpenicillin sodium for 3 days following the operation. After 3 weeks of postoperative recovery, rats subjected to right nephrectomy were fed a HFD (10% lard, 20% sucrose, 2.5% cholesterol, and 1% sodium cholate) supplied by Beijing Keao Xieli Feed Co., Ltd., while rats in the SC group were fed normal chow. After 4 weeks of dietary intervention, rats were intraperitoneally injected with 1% STZ (35 mg/kg). Rats in the SC group received an equal volume of sodium citrate-hydrochloric acid buffer solution. One week after STZ injection, an oral glucose tolerance test (OGTT) was carried out, and 24 h urine samples were collected. Rats with FBG levels ≥11.1 mmol/L and mAlb/Ucr ≥30 μg/mg (Tesch and Allen, 2007; Ghasemi et al., 2014; Matsui et al., 2017) were considered to have DN. The DN rats were divided into five groups using a random number table: The DN model group (DNM, n = 8), metformin group (MET, n = 8), low-dose HDB group (HDBL, n = 8), medium-dose HDB group (HDBM, n = 8) and high-dose HDB group (HDBH, n = 8).

### Drug Interventions

**FIGURE 1:**
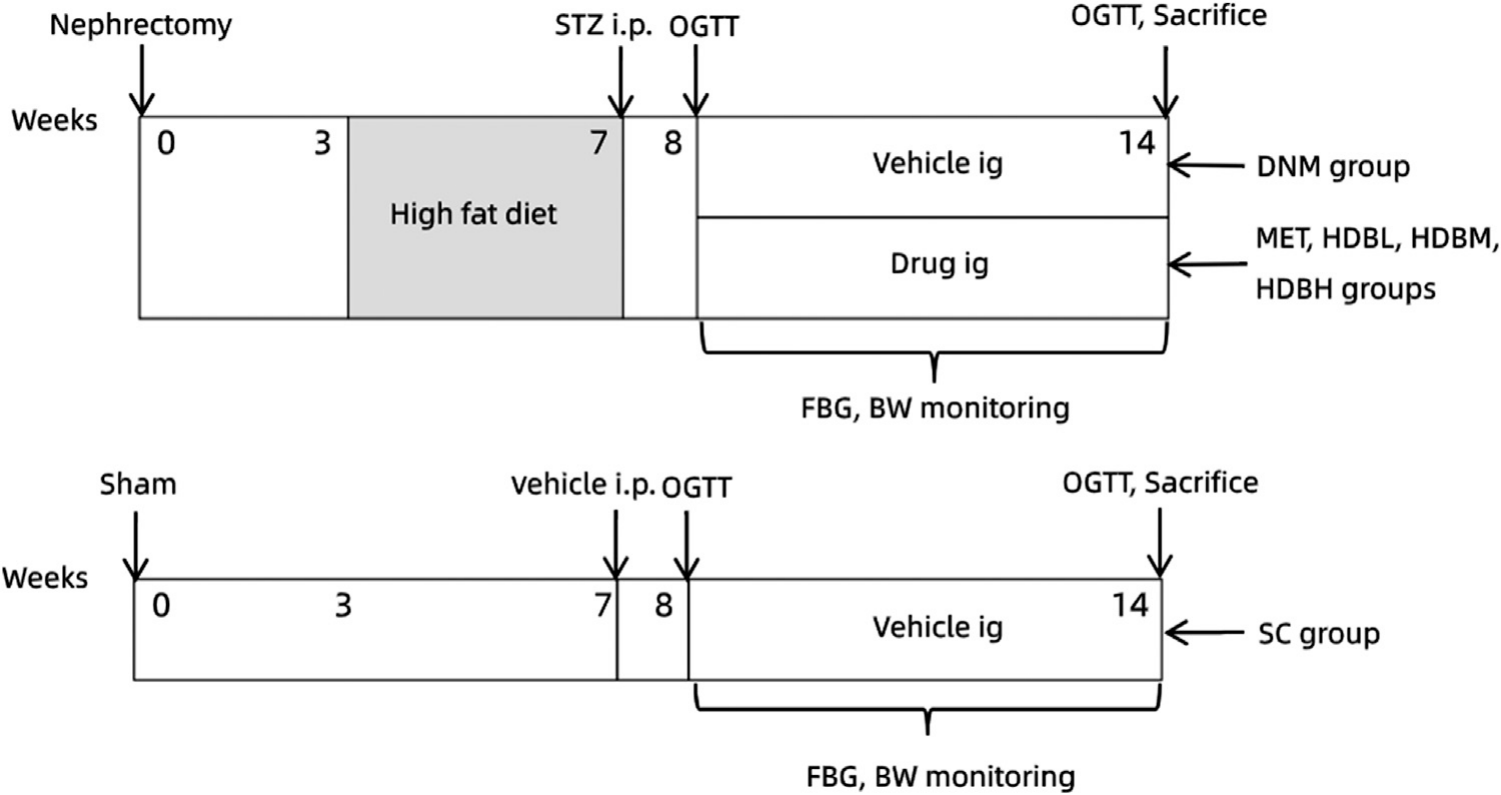
Experimental design scheme.Rats were subjected to unilateral nephrectomy (defined as 0 weeks), and three weeks later, they were fed a HFD for 4 weeks and then intraperitoneally injected (i.p.) with STZ (35 mg/kg). One week after STZ injection, an oral glucose tolerance test (OGTT) was carried out. The successfully modeled DN rats were divided into five groups: DN model group (DNM), metformin group (MET), low-dose HDB group (HDBL), medium-dose HDB group (HDBM) and high-dose HDB group (HDBH). The drug was administered intragastrically (i.g.) for 6 weeks. Rats in the sham operation control (SC) group were exposed at the right kidney during the operation, fed normal chow, and injected and gavaged with an equal volume of vehicle synchronously.

The original therapeutic instructions (Yang, et al., 2020) for the use of HDB in adults (70 kg body weight, BW) are as follows: 50 g crude drug per day for newly diagnosed patients, 60 g per day for mid-term patients, and 80 g per day for seriously ill patients should be administered. One period for the above treatment was 30, 40, or 50 days. By referring to the fourth edition of the Experimental Methodology of Pharmacology (Wei Wei, editor-in-chief), the equivalent dose was calculated according to the body surface area for each species. The corresponding doses for rats were 4.5, 5.4, and 7.2 g/kg/day. Based on the extraction rate of crude HDB (2.54%), the rats in the HDBL, HDBM, and HDBH groups were treated with 110mg/kg, 140mg/kg, 180mg/kg respectively. According to the clinical adult dosage of MET, rats in the MET group were treated with a dose of 45 mg/kg/day All drugs were dissolved in 0.5% sodium carboxymethyl cellulose (CMC-Na) and were given to rats intragastrically (i.g., 1 ml/100 g BW). Rats in the SC and DNM groups were gavaged with an equal volume of vehicle at a fixed time every day for 6 days per week with one day of rest. The drug intervention lasted 6 weeks. The experimental design is shown in Figure 1.

### Measurements

BW and FBG were monitored every week. OGTT was carried out before and after the drug intervention as follows: with free access to water, the rats were fasted for 12 h and gavaged with 50% glucose (2 g/kg). Blood glucose was measured by tail tip sampling at 0, 30, 60, 90, and 120 min. The area under the time-glucose curve (AUC) was calculated with the approximate trapezoidal area method (Eq. 1), where BG represents the blood glucose level at different time points after glucose burden. Urine was collected for 24 h before and after medication was administered. Urine was centrifuged at 2,000 r/min for 10 min at 4°C prior to the measurement of microalbumin (mAlb) and urine creatinine (Ucr). Blood samples were drawn from heart after intraperitoneally anesthetizing the rats with ketamine (3 mg/kg) and then centrifuged at 10,000 r/min for 15 min at 4°C to obtain serum for measuring serum creatinine (Scr) and serum urea nitrogen (BUN). All blood and urine indices were analyzed with a fully automatic biochemical analyzer. Animals were sacrificed after blood collection, and then the left kidney was removed by cesarean section and weighed to calculate the kidney index (KI) (Eq. 2). Half of the kidney was snap-frozen in liquid nitrogen, and the other half was processed for histological observation.

AUC=[(BG0+BG30)×0.5/2]+[(BG30+BG60)×0.5/2]+[(BG60+BG90)×0.5/2]+[(BG90+BG120)×0.5/2] (1)

KI=(left kidney mass/body mass)×100% (Li et al., 2010) (2)

### Histopathology

The kidney tissue was fixed in 10% neutral buffered formalin at 4°C for 48 h and then processed and embedded in paraffin. Paraffin-embedded kidney tissue was sectioned at a thickness of 4 μm, stained with periodic acid, and then incubated with a stock solution of methenamine. Thirty glomeruli in each section were randomly selected to observe the pathological alterations under a light microscope.

### Electron Microscopy Observation

The renal cortex was trimmed into 1 mm^3^ tissue blocks, fixed in 3.1% glutaraldehyde for 2 h, postfixed in 1.0% osmium tetroxide for 1 h, and embedded in Epon resin. The semithin sections were prepared for accurate localization. Ultrathin sections at a thickness of 60 nm were double stained with uranyl acetate and lead citrate and observed under TEM. At least 10 electron micrographs of glomeruli per rat were randomly taken at a magnification of 12,500× to observe the ultrastructural changes of podocytes and the GBM. The mean thickness of the GBM (TGBM) was calculated using measurements of a straight perpendicular line from the internal to the external layers of the GBM. Twenty different sites were chosen in all micrographs, and TGBM was expressed as the fold change based on the SC group.

### Western Blotting Analysis

Total protein from the renal cortex or cultured podocytes was extracted, and the protein concentrations were measured using an enhanced BCA kit. Protein lysates were separated by 10% SDS-PAGE and transferred onto a PVDF transfer membrane. Membranes were blocked with 5% skim milk in Tris-buffered saline containing Tween 20 (0.05%) for 2 h at room temperature and subsequently incubated with primary antibody (Nox4, 1:500 dilution; nephrin, 1:500 dilution) overnight at 4°C. After incubation with an HRP-conjugated secondary antibody (1:1,000 dilution) for 2 h at room temperature, the blots were observed using BeyoECL Plus according to the manufacturer’s protocol. The relative expression of protein was normalized according to β-actin expression. The densitometric analysis was performed using an Image Pro Plus software and expressed as the fold expression compared to the control (SC).

### Malondialdehyde Assay

Malondialdehyde (MDA), as a product of lipid peroxidation, was measured by thiobarbituric acid-reactive substances (T-BARS) methods using a lipid peroxidation MDA assay kit. Briefly, TBA solution was added to kidney tissue homogenate samples and standards, incubated at 90°C for 60 min, cooled in an ice bath for 10 min, transferred to microplate wells, and analyzed with a multiscan spectrum. Kidney tissue protein was estimated using an enhanced BCA kit, and the content was expressed as nmol malondialdehyde/mg protein.

### Immunofluorescence of Paraffin Sections

Paraffin slides of kidney tissue were dewaxed by an ethanol gradient. Antigens were retrieved using ethylenediamine tetraacetic acid (EDTA, pH 8.0) and subsequently blocked with 3% BSA in PBS for 30 min. Incubation with antibodies against Nox4 (1:100 dilution) or nephrin (1:100 dilution) was performed overnight at 4°C, followed by secondary antibody incubation with goat anti-rabbit IgG DyLight 488 for Nox4 and goat anti-mouse IgG (H&L) Cy3 for nephrin for 50 min at room temperature in a dark room. Photographs were acquired using a digital fluorescence microscope.

### Immunohistochemistry of Paraffin Sections

3-μm paraffin section of renal tissue was devaxed in xylene and rehydrated with graded ethanol. An antigen retrieval was performed with EDTA solution (pH 9.0) for 20 min at 100°C. The sections were blocked with 3% bovine serum albumin for 30 min at room temperature and then incubated with primary antibodies for WT1 (1:300) overnight at 4°C. The endogenous peroxidase was inactivated with 3% hydrogen peroxide by incubating the sections avoid light for 20 min at room temperature. After that, sections were incubated with secondary antibody and labeled with horseradish peroxidase. Labels were visualized with diaminobenzidine and nuclei were counterstained with hematoxylin. Staining was photographed under light microscopy and analyzed with an Image Pro Plus software.

### Podocyte Counting

Podocyte counting was performed on the abovementioned sections immunostained with WT1, a marker of podocyte nuclei, which was brown stained. 50 consecutive sections were photographed under light microscopy, and the images were analyzed morphometrically with ImagePro Plus software. The average glomerular volume was estimated with the Weibel formula, and the estimation of podocyte counts per glomerulus was determined by the stereological method proposed by Weibel (Weibel, 1981). And the podocyte cell number was calculated by multiplying the podocyte volume density by the average glomerular volume (Steffes et al., 2001; Macconi et al., 2006).

### Cell Viability Assay

The activity of cells was determined by MTT assay. In brief, cells were seeded in 96-well plates, stabilized for 24 h and incubated with HG for 24, 48, 72 h. 2.5, 5, 10, 20, 40, 80% of drug-containing serum mentioned above were added to podocytes with NG for 48 h to study the cytotoxic effects on podocytes; the optimized concentration of drug-containing serum were added to podocytes for 1 h followed by HG for 48 h to study the therapeutic effects of HDB on podocytes. After that, the cells were incubated with MTT according to the manufacturer’s protocol, and the results were analyzed using a multiscan spectrum.

### Reactive Oxygen Species Detection

The generation of ROS in the podocytes was determined using a flow cytometry (FCM) assay via the intracellular oxidation of dichlorodihydrofluorescein diacetate (DCFH-DA). The cells were seeded in 6-well plates, stabilized for 24 h, pretreated with or without different kinds of drug-containing serum aforementioned for 1 h followed by HG or NG for 48 h. After that, the intracellular ROS generation was detected using the ROS Assay kit following the manufacturer’s protocol. In brief, the pretreated cells were rinsed and washed with cold PBS, and then the cells were incubated in serum-free RPMI-1640 supplemented with 2,7-DCFH-DA (10 mmol/L) or PBS (as blank) in the dark at 37°C for 30 min. The cells were harvested, washed twice with PBS, and resuspended in serum-free RPMI-1640 medium for the FCM assay. FCM was performed on a BD flow cytometer by FlowJo VX10 software.

### Statistical Analysis

Statistical analysis was performed using the SPSS 20.0 software package. All the experimental values are presented as the means ± SE; *p* values of 0.05 were considered to be significant. Treatment effects were evaluated by one-way ANOVA, and normality and variance homogeneity were explored before analysis. One-way ANOVA was used under an equal variance condition, or a rank sum test was used if equal variances were not assumed. Repeated measurement data (for example, OGTT results) were analyzed by a multi-factor analysis of variance (MANOVA) method. LSD pairwise comparison methods were used to compare the results after variance analysis.

## Results

### General Status of the Rats

Rats subjected to right nephrectomy were not in a good state, with unsmooth fur and hypoactivity. Rats in the SC group were also in a poor state despite only undergoing exposure without nephrectomy. The rats in a poor state recovered 2 weeks after the operation. Two days after the STZ injection, the rats suffered from polyphagia, polydipsia, polyuria, and fatigue with obvious weight loss. One rat in the HDBL group died in the third week of drug intervention.

### Fasting Blood Glucose Monitoring Results and OGTT Analysis

**FIGURE 2:**
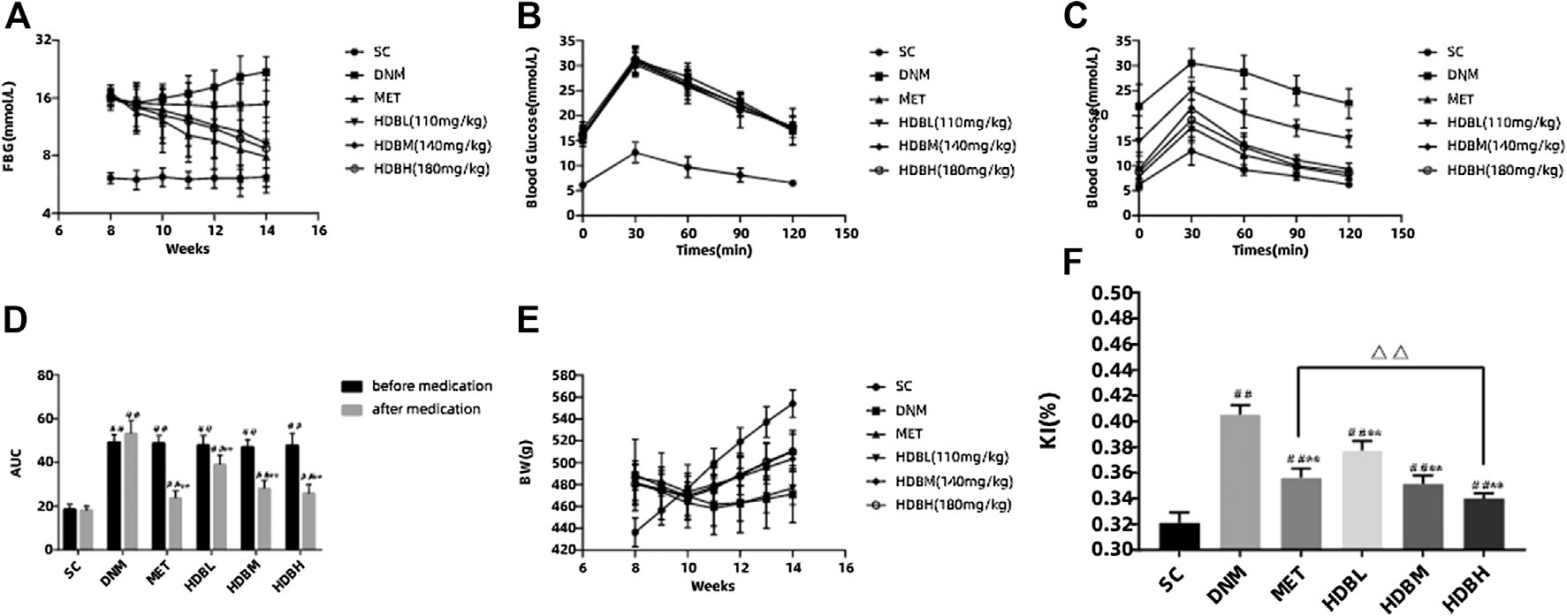
HDB reduced FBG, BW loss, proteinuria, and MDA level of DN rats. **(A)** Dynamic monitoring of FBG; **(B)** OGTT before medication; **(C)** OGTT after medication; **(D)** AUC before and after medication; **(E)** Dynamic monitoring of BW; **(F)** KI after medication administration; The data are expressed as the means ± SE for n = 8 rats/group, ^#^ *p* < 0.05, ^##^ *p* < 0.01 vs. SC, ^*^ *p* < 0.05, **p < 0.01 vs. DNM, ^△^ *p* < 0.05, ^△△^ *p* < 0.01 HDBH vs. MET. FBG, fasting blood glucose; OGTT, oral glucose tolerance test; AUC, area under the time-glucose curve; BW, body weight; KI, kidney index; mAlb, microalbumin; Ucr, urine creatinine; Scr, serum creatinine; BUN, blood urea nitrogen; MDA, malondialdehyde; SC, sham operation control group (vehicle); DNM, DN model group (vehicle); MET, metformin group (4.5 mg/ml); HDBL, low-dose HDB group (110 mg/kg); HDBM, medium-dose HDB group (140 mg/kg); HDBH, high-dose HDB group (180 mg/kg).

The DNM and drug administration groups had a much higher FBG and AUC than the SC group (^##^ *p* < 0.01). Both HDB and MET reduced the FBG and AUC in DN rats. After medication administration, there was no significant difference in the blood glucose level among the HDBH, HDBM and MET groups compared with the SC group (*p* > 0.05), but the AUC in the former three groups was still higher than that in the SC group (^##^ *p* < 0.01). The FBG and AUC in the drug administration groups were markedly reduced compared with those in the DNM group (**p < 0.01), while the hypoglycemic effect of HDBL was not as dramatic as that of the other treatments (Figures 2A–D).

### BW Monitoring and Kidney Index Calculation

Before medication administration, the rats in the DNM and drug administration groups had a much higher BW than the rats in the SC group due to HFD feeding (^##^ *p* < 0.01), while there was obvious weight loss after STZ injection. Dynamic monitoring of BW showed that HDB and MET could relieve weight loss in DN rats, and by the end of the experiment, the HDB and MET groups had a much higher BW than the DNM group (**p < 0.01) but a much lower BW than the SC group (^##^ *p* < 0.01). The KI of the DNM group was higher than that of the SC (^##^ *p* < 0.01) and drug administration groups (**p < 0.01), and both HDB and MET reduced the KI of the DN rats (Figures 2E,F).

### Urine and Blood Testing Results

Before medication, the mAlb/Ucr in the DNM and drug administration groups was markedly higher than that in the SC group (^##^ *p* < 0.01). After medication, the drug administration groups had lower mAlb/Ucr values compared with the DNM group (**p < 0.01) but higher values than the SC group (^##^ *p* < 0.01). The levels of Scr and BUN in the drug administration groups were also reduced compared with those in the DNM group (**p < 0.01), and there were no significant differences between the HDBH and SC groups (*p* > 0.05). HDBH had a better effect on reducing mAlb/Ucr, Scr and BUN levels than MET (^△△^ *p* < 0.01) (Table 1).

**TABLE 1:** Effect of HDB on mAlb/Ucr, Scr, BUN and MDA in DN rats (**‾**x ± s, n = 8).

Variable	Time	Group					
		SC	DNM	MET	HDBL	HDBM	HDBH
mAlb/Ucr (μg/mg)	Before medication	10.45 ± 1.40	36.54 ± 1.39^##^	37.93 ± 2.69^##^	37.53 ± 1.31^##^	37.19 ± 1.69^##^	38.39 ± 1.96^##^
	After medication	11.28 ± 1.53	58.27 ± 2.83^##^	21.22 ± 1.87^##**^	34.24 ± 1.52^##**^	22.81 ± 2.36^##**^	16.68 ± 1.08^##**^
BUN (mmol/L)	After medication	6.21 ± 0.48	14.63 ± 1.68^##^	9.04 ± 1.24^##**^	9.90 ± 2.41^##**^	8.84 ± 1.90^##**^	6.99 ± 1.82^**^
Scr (μmol/L)	After medication	32.22 ± 3.19	61.50 ± 4.24^##^	37.13 ± 2.10^#**^	39.18 ± 5.25^##**^	37.16 ± 3.38^#**^	33.10 ± 3.73^**^
MDA (nmol/mg)	After medication	3.80 ± 0.18	8.28 ± 0.53^##^	4.93 ± 0.56^##**^	5.67 ± 0.73^##**^	4.88 ± 0.38^##**^	4.12 ± 1.02^**^

### Malondialdehyde in Kidney Tissue

MDA is often used as a marker of lipid peroxidation and is essential to assay for the detection of oxidative damage in cells and tissues. Here, we found that HDB and MET could significantly reduce the content of MDA in the kidney, and the results showed that HDBH was more effective in anti-lipid peroxidation than MET (^△^ *p* < 0.05) (Table 1).

### Observations of PASM Staining

**FIGURE 3:**
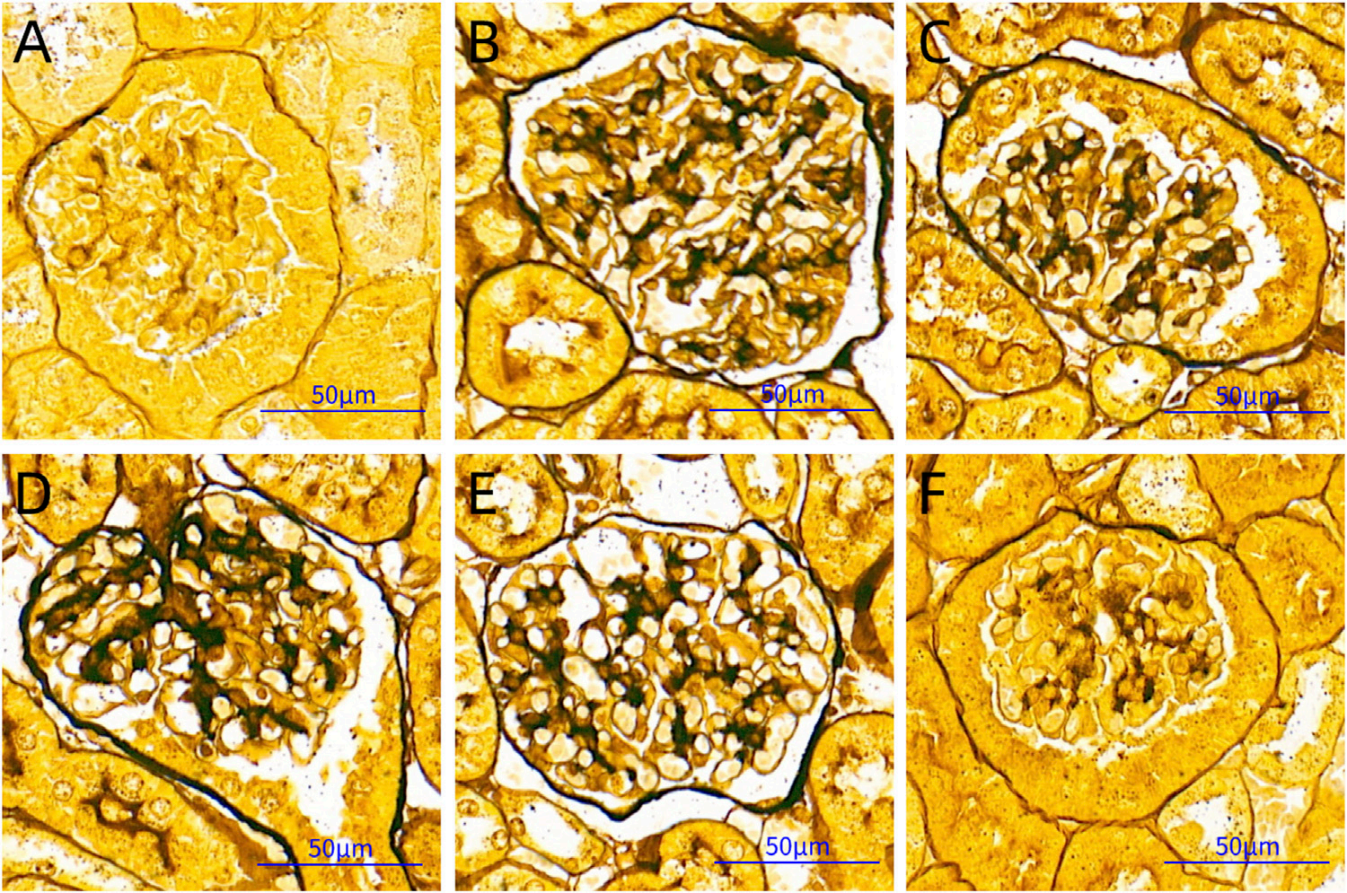
PASM staining pictures. Scale bar = 50 μm. **(A)** SC, sham operation control group (vehicle); **(B)** DNM, DN model group (vehicle); **(C)** MET, metformin group (45 mg/kg); **(D)** HDBL, low-dose HDB group (110 mg/kg); **(E)** HDBM, medium-dose HDB group (140 mg/kg); **(F)** HDBH, high-dose HDB group (180 mg/kg). PASM, periodic acid-silver methenamine.

GBM is rich in type IV collagen and can be stained black with periodic acid‐silver methenamine. Based on the black staining in the images, we found that there was obvious thickening of the GBM in the DNM group compared with the other groups. HDB and MET could significantly reduce the pathological changes of the GBM (Figure 3).

### Expression of Nox4 and Nephrin in Kidney

**FIGURE 4:**
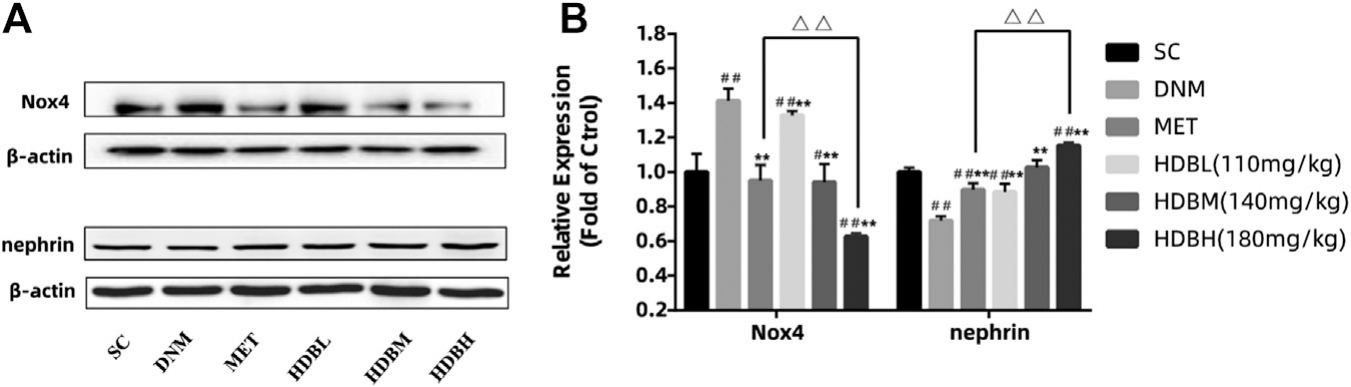
HDB downregulated Nox4 and nephrin expression. **(A)** Representative Western blotting bands of Nox4; **(B)** Representative Western blotting bands of nephrin; **(C)** Quantitative analysis of Western blotting expression of Nox4 and nephrin was controlled by β-actin. Nox4, a subtype of nicotinamide adenine dinucleotide phosphate (NADPH) oxidase; SC, sham operation control group (vehicle); DNM, DN model group (vehicle); MET, Metformin group (45 mg/kg); HDBL, low-dose HDB group (110 mg/kg); HDBM, medium-dose HDB group (140 mg/kg); HDBH, high-dose HDB group (180 mg/kg). The data are expressed as the fold change based on the SC group (n = 3 rats/group), ^#^ *p* < 0.05, ^##^ *p* < 0.01 vs. SC, **p < 0.01 vs. DNM, ^△△^ *p* < 0.01 HDBH vs. MET.

**FIGURE 5:**
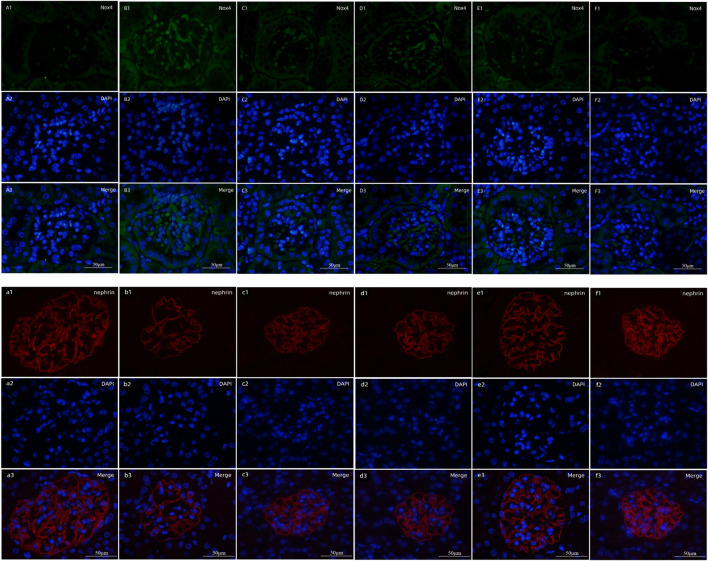
Fluorescence staining of Nox4 and nephrin. Scale bar = 50 μm. **(A1–F1)** Expression of Nox4 labeled by goat anti-rabbit IgG DyLight 488; **(A2–F2)** cell nuclei observed by DAPI; **(A3–F3)** merged images. **(A1–A3)** SC, sham operation control group (vehicle); **(B1–B3)** DNM, DN model group (vehicle); **(C1–C3)** MET, metformin group (45 mg/kg); **(D1–D3)** HDBL, low-dose HDB group (110 mg/kg); **(E1–E3)** HDBM, medium-dose HDB group (140 mg/kg); **(F1–F3)** HDBH, high-dose HDB group (180 mg/kg). **(a1–f1)** Expression of nephrin labeled by goat anti-mouse IgG (H&L) Cy3; **(a2–f2)** cell nuclei observed by DAPI; **(a3–f3)** merged images. **(a1–a3)** SC, sham operation control group (vehicle); **(b1–b3)** DNM, DN model group (vehicle); **(c1–c3)** MET, Metformin group (45 mg/kg); **(d1–d3)** HDBL, low-dose HDB group (110 mg/kg); **(e1–e3)** HDBM, medium-dose HDB group (140 mg/kg); **(f1–f3)** HDBH, high-dose HDB group (180 mg/kg).

Nox4, as the major producer of ROS in the kidney, demonstrated markedly higher expression in DNM (^##^ *p* < 0.01); nephrin, as a marker protein of podocytes, was decreased sharply in DNM (^##^ *p* < 0.01). HDB and MET significantly downregulated Nox4 expression but upregulated nephrin expression (**p < 0.01), and the results showed that HDBH was more effective in anti-oxidation than MET (^△△^ *p* < 0.01) (Figure 4). To verify the relative expression of Nox4 and nephrin, immunofluorescence staining of paraffin sections was performed. Nox4 was predominantly localized to glomeruli, as demonstrated by the increased fluorescence in the DNM group according to the goat anti-rabbit IgG DyLight 488 staining. Nephrin, as a marker protein of podocytes, was tested to detect podocyte loss. Images of nephrin labeled by goat anti-mouse IgG (H&L) Cy3 from DNM rats demonstrated an obvious attenuation of fluorescence. HDB and MET reduced podocyte loss, as demonstrated by the increased expression of nephrin, especially in the HDBH, HDBM, and MET groups (Figure 5).

### Podocyte Counting by WT1 Immunostaining

**FIGURE 6:**
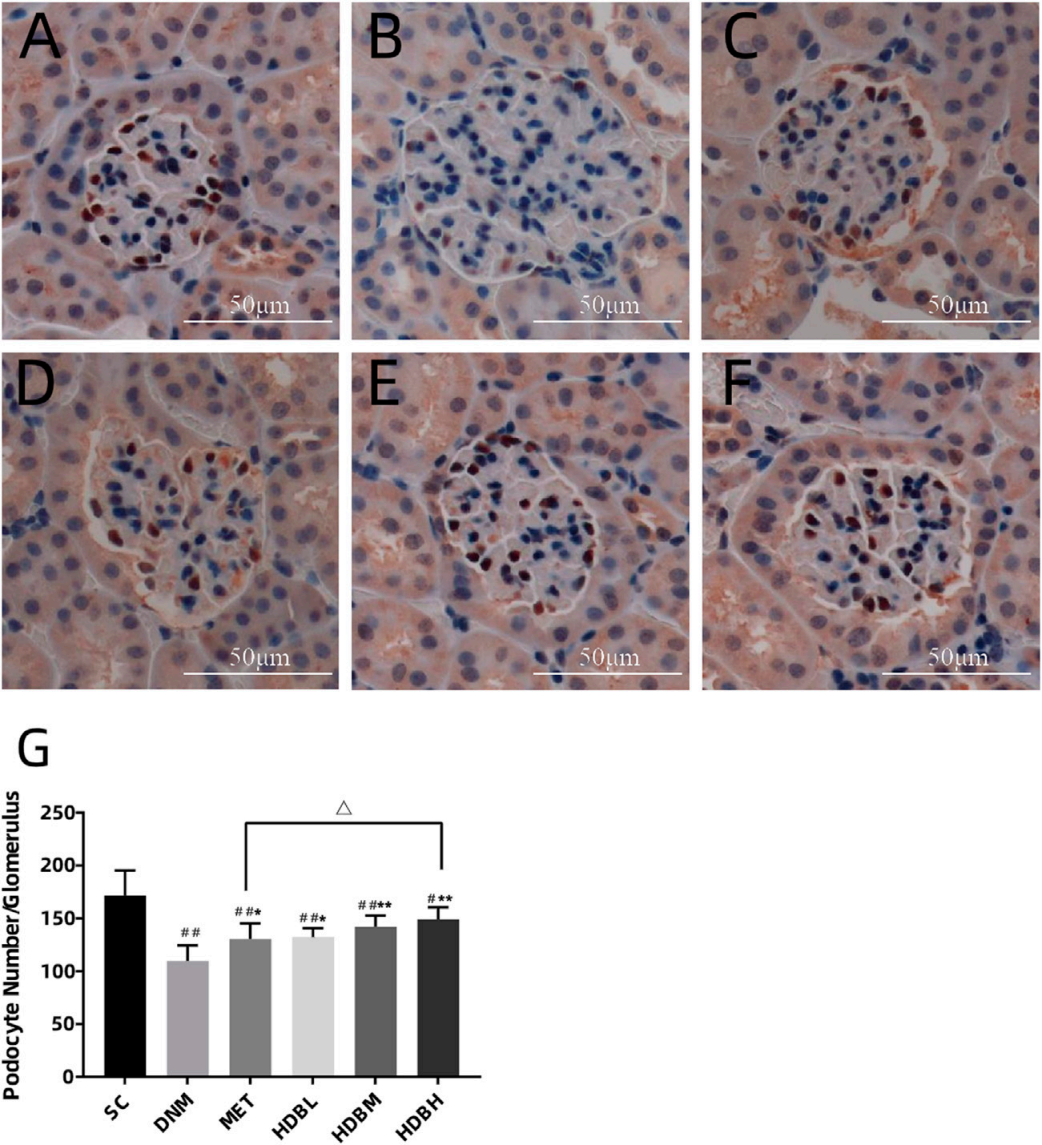
HDB reduced podocyte loss. Scale bar = 50 μm. **(A–F)** Representative images of WT1 immunostaining; **(G)** Podocyte counting. **(A)** SC, sham operation control group (vehicle); **(B)** DNM, DN model group (vehicle); **(C)** MET, metformin group (45 mg/kg); **(D)** HDBL, low-dose HDB group (110 mg/kg); **(E)** HDBM, medium-dose HDB group (140 mg/kg); **(F)** HDBH, high-dose HDB group (180 mg/kg). The data are expressed as the means ± SE for n = 8 rats/group, ^#^ *p* < 0.05, ^##^ *p* < 0.01 vs. SC, ^*^ *p* < 0.05, **p < 0.01 vs. DNM, ^△^ *p* < 0.05 HDBH vs. MET.

Podocyte counting by WT1 immunostaining showed that, podocyte number decreased markedly in the DNM group compared with the SC group (^##^ *p* < 0.01), and both HDB and MET could reduced podocyte loss (^*^ *p* < 0.05, or **p < 0.01), especially in the HDBH group, a significant increase of podocytes was observed compared with the MET group (^△^ *p* < 0.05) (Figure 6).

### Ultrastructure Changes Under TEM

**FIGURE 7:**
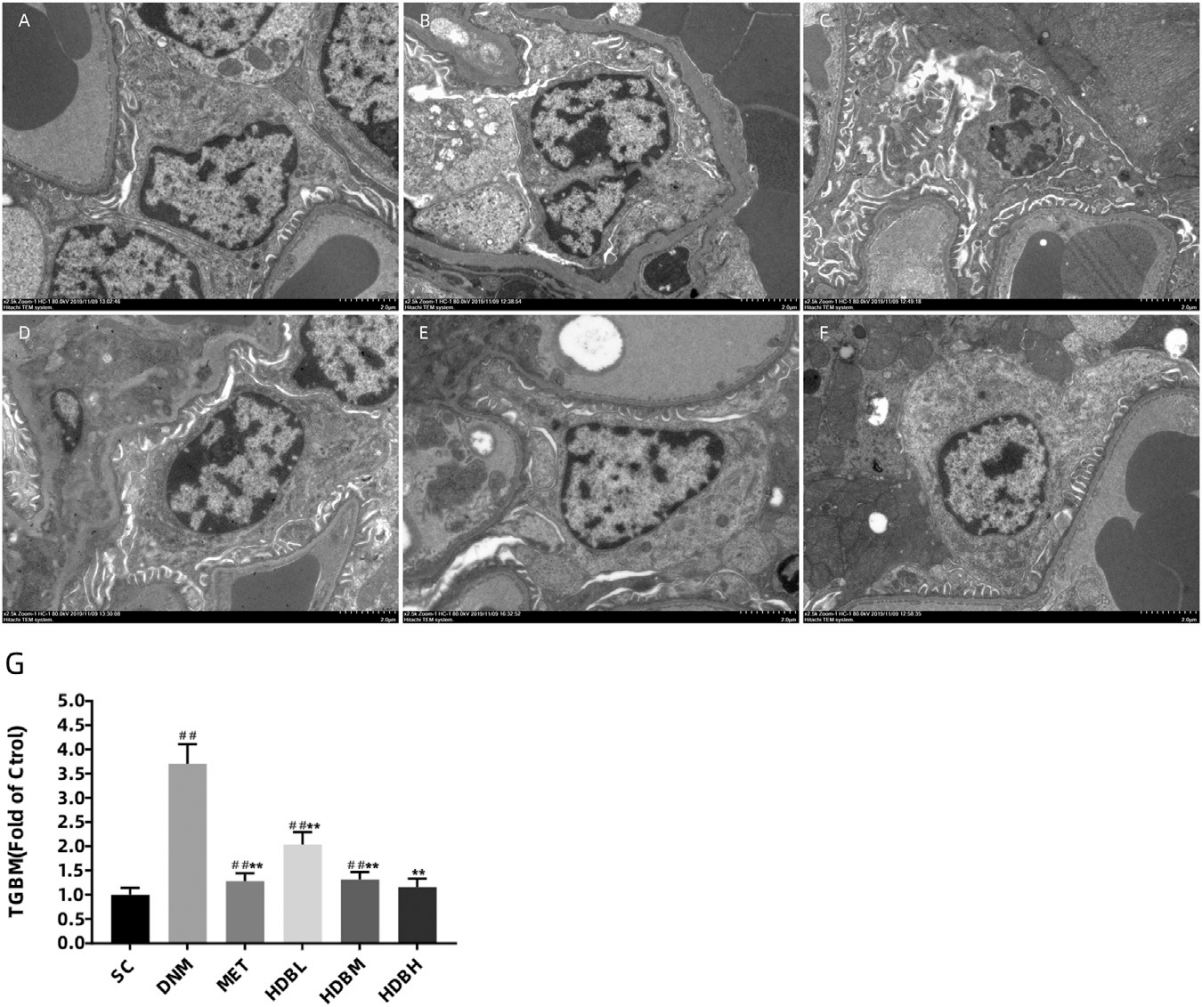
HDB reduced foot process fusion and GBM damage in DN rats. Scale bar = 2 μm. Podocyte injury and GBM changes were observed by TEM **(A–F)**. **(A)** SC, sham operation control group (vehicle); **(B)** DNM, DN model group (vehicle); **(C)** MET, metformin group (45 mg/kg); **(D)** HDBL, low-dose HDB group (110 mg/kg); **(E)** HDBM, medium-dose HDB group (140 mg/kg); **(F)** HDBH, high-dose HDB group (180 mg/kg). **(G)** TGBM comparison. TGBM, mean thickness of the GBM. The data are expressed as the fold change based on the sham operation control group (n = 3 rats/group), ^##^ *p* < 0.01 vs. SC, **p < 0.01 vs. DNM.

Podocyte injury and GBM changes were observed by TEM to further confirm the above results, especially the loss of podocytes demonstrated by fluorescence staining of nephrin. As shown in the image, the GBM of SC rats was clear and intact, and podocytes were also observed to show good integrity. In contrast, irregular thickening of the GBM and foot process fusion was found in DNM rats (^##^ *p* < 0.01). Furthermore, the mitochondrial cristae were unclear, even vacuolization occurred in DNM rats. Apparent improvements in GBM thickening and foot process fusion were observed in the drug administration groups (**p < 0.01). TGBM measurements showed further evidence for the observations above, especially in the HDBH group, and no significant difference was found compared to the SC group (*p* > 0.05) (Figure 7).

### Huidouba Protects Against HG-Induced Podocyte Apoptosis

**FIGURE 8:**
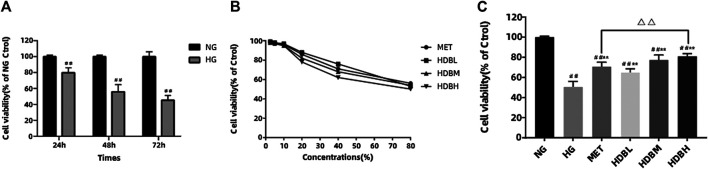
HDB reduced HG-induced podocytes apoptosis. **(A)** The podocytes were incubated with HG (30 mmol/L) for 24, 48, 72 h, and the cell viability were examined using MTT assay; **(B)** Different concentrations (2.5, 5, 10, 20, 40, 80%) of drug-containing serum were added to the NG-treated podocytes, and the cytotoxic effects were evaluated using MTT assay; **(C)** Different kinds of drug-containing serum were added to the NG or HG-treated podocytes, and the anti-apoptosis effects were detected using MTT assay. The data are expressed as the means ± SE, ^##^ *p* < 0.01 vs. NG, **p < 0.01 vs. HG, ^△△^ *p* < 0.01 HDBH vs. MET. MTT, Thiazolyl Blue Tetrazolium Bromide; NG, normal glucose-treated group; HG, high glucose-induced group; MET, Metformin-containing serum treated group (45 mg/kg); HDBL, low-dose HDB-containing serum treated group (110 mg/kg); HDBM, medium-dose HDB-containing serum treated group (140 mg/kg); HDBH, high-dose HDB-containing serum treated group (180 mg/kg).

In order to examine the effect of HG on cell survival, MTT assay was performed. Compared with the NG group, HG reduced the cell viability in a time-dependent manner (^##^ *p* < 0.01) (Figure 8A). We also tested the cytotoxicity of different concentrations of drug-containing serum on podocytes, and the cells retained almost the same viability when incubated with concentrations of 2.5–10% for 48 h, whereas concentrations >20% markedly altered the cell viability (Figure 8B). Therefore, we chose 10% concentration to study the curative effect, and there is no obvious cytotoxicity. HDB and MET-containing serum could significantly increase the cell viability compared with the HG group (**p < 0.01), and the results indicated that high-dose HDB-containing serum had a better effect than MET-containing serum on reducing HG-induced podocytes apoptosis (^△△^ *p* < 0.01) (Figure 8C).

### Huidouba Inhibits the HG-Induced Reactive Oxygen Species Generation by Downregulated Nox4 Expression in Podocytes

**FIGURE 9:**
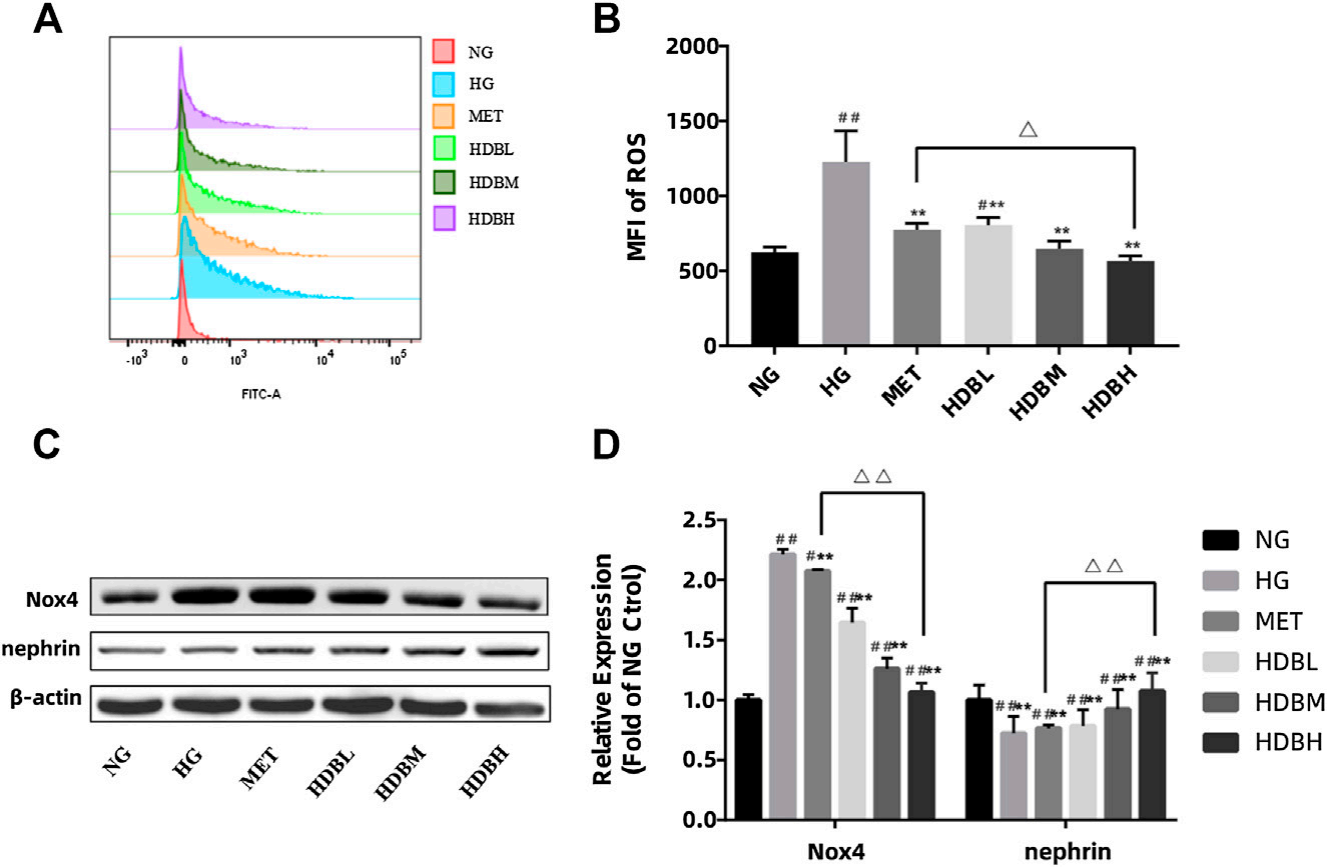
HDB inhibits the HG-induced ROS generation by downregulated Nox4 expression in podocytes. **(A,B)** The production of ROS was detected by flow cytometry following treatment with different kinds of drug-containing serum under NG or HG for 48 h. **(C)** Representative Western blotting bands of Nox4 and nephrin; **(D)** Quantitative analysis of Western blotting expression of Nox4 and nephrin was controlled by β-actin. The data are expressed as the means ± SE, ^#^ *p* < 0.05, ^##^ *p* < 0.01 vs. NG, **p < 0.01 vs. HG, ^△^ *p* < 0.05 ^△△^ *p* < 0.01 HDBH vs. MET. ROS, reactive oxygen species; MFI, mean fluorescence intensity; Nox4, a subtype of nicotinamide adenine dinucleotide phosphate (NADPH) oxidase; NG, normal glucose-treated group; HG, high glucose-induced group; MET, Metformin-containing serum treated group (45 mg/kg); HDBL, low-dose HDB-containing serum treated group (110 mg/kg); HDBM, medium-dose HDB-containing serum treated group (140 mg/kg); HDBH, high-dose HDB-containing serum treated group (180 mg/kg).

To gain an insight into the mechanism of podocyte apoptosis, we measured the generation of ROS induced by HG. Compared with the NG group, the amount of ROS generation in the HG group increased significantly. (##p < 0.01) HDB and MET-containing serum reduced HG-induced intracellular ROS (**p < 0.01) (Figures 9A,B). Moreover, the HDBH group had a better effect on reducing ROS production in podocytes (△p < 0.05). As mentioned above, Nox4 is the main source of ROS in podocytes and nephrin is essential to maintain the normal filtration barrier of the kidney, in order to verify that HDB reduces podocyte damage by antioxidant action, we examined Nox4 and nephrin expression in HG-treated podocytes. As shown in Figures 9C,D, compared with the NG group, podocytes treated with HG exerted a marked increase in Nox4 expression, whereas a decrease in nephrin (^##^ *p* < 0.01). HDB and MET-containing serum significantly reversed these results compared with the HG group (**p < 0.01), and the HDBH group showed a much better effects than the MET group (^△△^ *p* < 0.01).

## Discussion

Frank C. Brosius et al. found that diet has an apparent effect on DN (Brosius 3rd et al., 2009). The rats used for model establishment were fed a HFD 3 weeks after right nephrectomy. The DN rats showed apparent increases in weight gain compared with the SC rats 2 weeks after HFD feeding, whereas in the fourth week of the diet intervention, the rats ate less than normal and became reluctant to consume the HFD, so the HFD was changed to normal chow the following week. It has been reported that nongenetic DN rat models often show different levels of β cell failure induced by STZ injection (Srinivasan and Ramarao, 2007; Almalki, 2019). The American Diabetes Complications Consortium (AMDCC, http://www.amdcc.org) recommended low-dose STZ for DN establishment, so we adopted low-dose STZ (35 mg/kg) injection to induce DN. However, we observed a reduction in weight over the next 3 weeks following STZ injection, so the possibility of acute injury to β cells was not excluded. Notably, by the end of the experiment, the level of FBG in the HDBH, HDBM, and MET groups showed no difference compared with that in the SC group; however, the increase in the AUC compared with that in the SC group indicated that there was still impaired glucose tolerance in these groups.

HDB, as a folk medicine, is widely used for the effective treatment of DM and its complications. Previous studies focused on the basic research about pharmacodynamic substances of HDB, HDB polysaccharides (Wu et al., 2013) and HDB protein (Peng and Li 2010) were found effective on hypoglycemic activity. As we known, HDB has been used by water decocting method in the Mount Emei area of Sichuan Province for generations. In consideration of the integrity of Traditional Chinese medicine and the complexity of the mechanism of action, we followed the local medication regimen, using aqueous extract to study the therapeutic effects of HDB on the treatment of DN. In our experiment, HDB was purchased from its origin in the Mount Emei area in Sichuan Province. We used an aqueous extract and designed the HDBH, HDBM, and HDBL groups according to the original therapeutic reference. Consistent with previous studies, an obvious hypoglycemic effect was observed in DN rats, but beyond that, an antioxidative property of HDB was discovered.The results showed that high-dose HDB was even more effective than MET in reducing proteinuria and antioxidation, and no obvious hepatotoxicity and nephrotoxicity were observed in our previous study, data were not shown; however, a low dose of HDB was not as effective as the higher doses.

Proteinuria caused by podocyte injury and a decreased glomerular filtration rate (GFR) are two important manifestations that occur during the progression of DN into ESRD (Müller-Deile et al., 2018). Studies have shown that there are already different levels of podocyte impairment in the early stage of DN. Therefore, scientists should pay more attention to podocyte damage in DN development (Liu et al., 2018; Toffoli et al., 2018). Podocytes, as visceral epithelial cells attached to the lateral basement membrane of the glomerulus, are involved in forming the final barrier of the glomerular filtration membrane. Nephrin, as the main component of the slit diaphragm, is significantly less expressed in DN patients, and the degree of reduction in expression is positively correlated with proteinuria development (Ma et al., 2018). In this study, we found decreased nephrin expression in both DN rats and HG-induced podocytes, and a significant decrease of podocyte number was observed in the DNM group, which may indicate podocyte loss in the progression of DN. HDBH, HDBM and MET reduced podocyte loss and upregulated nephrin expression. TEM observations confirmed the findings.

As multiple studies have reported, a persistent high-glucose environment may induce oxidative stress and accelerate cell apoptosis by inducing excess ROS production (Diaz-Morales et al., 2016; Rovira-Llopis et al., 2017). The elevated production of ROS plays a key role in the development and progression of DN (Ma et al., 2013; Chen et al., 2015). In this study, we found that HG-induced ROS generation in cultured podocytes, HDB and MET could markedly reduce the level of intracellular ROS. Lipid peroxidation is a well-established marker associated with cellular oxidative injury, and MDA is frequently used as an indicator of oxidative stress. The current results indicated that lipid peroxidation was significantly increased in DN rats, while HDB and MET could markedly reduce the level of MDA in kidney tissue. Nicotinamide adenine dinucleotide phosphate (NADPH) oxidase (Nox), as the major inducer of oxidative stress in different cell types, has seven subtypes, Nox1-5, Duox1 and Duox2 (Drummond et al., 2011). Nox1, Nox2 and Nox4 have been reported to be mainly expressed in the renal cortex (Bedard and Krause, 2007), and Nox4 is predominantly expressed in the kidney (Sedeek et al., 2010). Studies have shown that glucose can activate Nox4 and then induce cell apoptosis in cultured podocytes and DM rats (Eid et al., 2009, 2010; Jha et al., 2016). To explore the factors associated with podocyte damage, we examined Nox4 expression in the kidneys of DN rats and HG-induced podocytes, and found markedly upregulated Nox4 expression. HDBH, HDBM and MET were associated with significantly downregulated Nox4 expression and protected DN rats from podocyte oxidative damage and proteinuria.

Interestingly, we found obvious mitochondrial cristae rupture under TEM, even vacuolization occurred in DNM. It has been reported that mitochondrial dynamics play an important role in type 2 DM and its complications. An imbalance in mitochondrial dynamics may induce mitochondrial dysfunction, reduce ATP and mitochondrial DNA (mtDNA) synthesis, and cause the loss of the mitochondrial membrane potential (MMP); subsequently, podocyte apoptosis occurs via the mitochondrial apoptotic pathway, which may finally contribute to the development of DN (Rovira-Llopis et al., 2017). Moreover, Nox4 has been reported to mainly localize in mitochondria and is the major source of ROS in podocytes (Das et al., 2014). This strongly suggests that ROS originating from Nox4 may be the initial cause of podocyte oxidative stress, which induces an imbalance in mitochondrial dynamics and finally results in apoptosis. Taken together, these data will provide extremely valuable clues for us to explore the mechanism of HDB in treating DM and its complications.

## Data Availability Statement

The raw data supporting the conclusions of this article will be made available by the authors, without undue reservation.

## Ethics Statement

The animal study was reviewed and approved by The Laboratory Animal Ethics Committee of Chengde Medical University.

## Author Contributions

KY, CY, and ZP conceived and designed the study, KY and CY wrote the manuscript. KY, YB, and NY carried out the animal experiments and helped to revise the manuscript. BL and GH analyzed and interpreted the data. All authors contributed to manuscript revision, read, and approved the submitted version.

## Funding

The study was supported by the National Natural Science Foundation of China (81804217); Key project of Chengde Medical University (201810); Major scientific project of Chengde Medical University (KY2020006).

## Conflict of Interest

The authors declare that the research was conducted in the absence of any commercial or financial relationships that could be construed as a potential conflict of interest.

## References

[B1] Almalki D. A. (2019). Renoprotective effect of *Ocimum Basilicum* (basil) against diabetes-induced renal affection in albino rats. Mater. Sociomed. 31, 236–240. 10.5455/msm.2019.31.236-240 32082085 PMC7007616

[B2] Bedard K. Krause K. H. (2007). The NOX family of ROS-generating NADPH oxidases: physiology and pathophysiology. Physiol. Rev. 87, 245–313. 10.1152/physrev.00044.2005 17237347

[B3] Brosius F. C. 3rd Alpers C. E. Bottinger E. P. Breyer M. D. Coffman T. M. Gurley S. B. (2009). Mouse models of diabetic nephropathy. J. Am. Soc. Nephrol. 20, 2503–2512. 10.1681/ASN.2009070721 19729434 PMC4075053

[B4] Chen J. Chen J. K. Harris R. C. (2015). EGF receptor deletion in podocytes attenuates diabetic nephropathy. J. Am. Soc. Nephrol. 26, 1115–1125. 10.1681/ASN.2014020192 25185988 PMC4413759

[B5] Das R. Xu S. Quan X. Nguyen T. T. Kong I. D. Chung C. H. (2014). Upregulation of mitochondrial Nox4 mediates TGF-β-induced apoptosis in cultured mouse podocytes. Am. J. Physiol. Ren. Physiol. 306, F155–F167. 10.1152/ajprenal.00438.2013 24259511

[B6] Diaz-Morales N. Rovira-Llopis S. Bañuls C. Escribano-Lopez I. de Marañon A. M. Lopez-Domenech S. (2016). Are mitochondrial fusion and fission impaired in leukocytes of type 2 diabetic patients. Antioxidants Redox Signal. 25, 108–115. 10.1089/ars.2016.6707 27043041

[B7] Drummond G. R. Selemidis S. Griendling K. K. Sobey C. G. (2011). Combating oxidative stress in vascular disease: NADPH oxidases as therapeutic targets. Nat. Rev. Drug Discov. 10, 453–471. 10.1038/nrd3403 21629295 PMC3361719

[B8] Eid A. A. Ford B. M. Block K. Kasinath B. S. Gorin Y. Ghosh-Choudhury G. (2010). AMP-activated protein kinase (AMPK) negatively regulates Nox4-dependent activation of p53 and epithelial cell apoptosis in diabetes. J. Biol. Chem. 285, 37503–37512. 10.1074/jbc.M110.136796 20861022 PMC2988355

[B9] Eid A. A. Gorin Y. Fagg B. M. Maalouf R. Barnes J. L. Block K. (2009). Mechanisms of podocyte injury in diabetes: role of cytochrome P450 and NADPH oxidases. Diabetes 58, 1201–1211. 10.2337/db08-1536 19208908 PMC2671039

[B10] Ghasemi A. Khalifi S. Jedi S. (2014). Streptozotocin-nicotinamide-induced rat model of type 2 diabetes (review). Acta Physiol. Hung. 101, 408–420. 10.1556/APhysiol.101.2014.4.2 25532953

[B11] Jha J. C. Thallas-Bonke V. Banal C. Gray S. P. Chow B. S. Ramm G. (2016). Podocyte-specific Nox4 deletion affords renoprotection in a mouse model of diabetic nephropathy. Diabetologia 59, 379–389. 10.1007/s00125-015-3796-0 26508318 PMC6450410

[B12] Li S. F. Wang Y. R. Wang Y. C. Hu Q. Zhao Y. (2010). Comparison different model construction methods of diabetic nephropathy SD rats. Acta Univ. Med. Nanjing 30, 1123–1128. 10.1360/972010-923

[B13] Liu T. Chen X. M. Sun J. Y. Jiang X. S. Wu Y. Yang S. (2018). Palmitic acid-induced podocyte apoptosis via the reactive oxygen species-dependent mitochondrial pathway. Kidney Blood Press. Res. 43, 206–219. 10.1159/000487673 29490300

[B14] Ma T. Zhu J. Chen X. Zha D. Singhal P. C. Ding G. (2013). High glucose induces autophagy in podocytes. Exp. Cell Res. 319, 779–789. 10.1016/j.yexcr.2013.01.018 23384600 PMC3628680

[B15] Ma Y. Yang Q. Zhong Z. Liang W. Zhang L. Yang Y. (2018). Role of c-Abl and nephrin in podocyte cytoskeletal remodeling induced by angiotensin II. Cell Death Dis. 9, 185 10.1038/s41419-017-0225-y 29416010 PMC5833834

[B16] Macconi D. Bonomelli M. Benigni A. Plati T. Sangalli F. Longaretti L. (2006). Pathophysiologic implications of reduced podocyte number in a rat model of progressive glomerular injury. Am. J. Pathol. 168, 42–54. 10.2353/ajpath.2006.050398 16400008 PMC1592676

[B17] Marathe P. H. Gao H. X. Close K. L. (2017). American diabetes association standards of medical Care in diabetes 2017. J. Diabetes 9, 320–324. 10.1111/1753-0407.12524 28070960

[B18] Matsui T. Higashimoto Y. Nishino Y. Nakamura N. Fukami K. Yamagishi S. I. (2017). RAGE-aptamer blocks the development and progression of experimental diabetic nephropathy. Diabetes 66, 1683–1695. 10.2337/db16-1281 28385802

[B19] Müller-Deile J. Schröder P. Beverly-Staggs L. Hiss R. Fiedler J. Nyström J. (2018). Overexpression of preeclampsia induced microRNA-26a-5p leads to proteinuria in zebrafish. Sci Rep 8, 3621 10.1038/s41598-018-22070-w 29483572 PMC5827519

[B20] Peng L. Li Z. M. (2010). Hypoglycemic effect of Huidouba on streptozotocin-induced diabetic rats. Lishizhen Med. Mater. Med. Res. 21 (12), 3060–3061. 10.3969/j.issn.1008-0805.2010.12.011

[B21] Rovira-Llopis S. Bañuls C. Diaz-Morales N. Hernandez-Mijares A. Rocha M. Victor V. M. (2017). Mitochondrial dynamics in type 2 diabetes: pathophysiological implications. Redox Biol. 11, 637–645. 10.1016/j.redox.2017.01.013 28131082 PMC5284490

[B22] Sanz M. Ceriello A. Buysschaert M. Chapple I. Demmer R. T. Graziani F. (2018). Scientific evidence on the links between periodontal diseases and diabetes: consensus report and guidelines of the joint workshop on periodontal diseases and diabetes by the International Diabetes Federation and the European Federation of Periodontology. J. Clin. Periodontol. 45, 138–149. 10.1111/jcpe.12808 29280174

[B23] Sedeek M. Callera G. Montezano A. Gutsol A. Heitz F. Szyndralewiez C. (2010). Critical role of Nox4-based NADPH oxidase in glucose-induced oxidative stress in the kidney: implications in type 2 diabetic nephropathy. Am. J. Physiol. Ren. Physiol. 299, F1348–F1358. 10.1152/ajprenal.00028.2010 20630933

[B24] Srinivasan K. Ramarao P. (2007). Animal models in type 2 diabetes research: an overview. Indian J. Med. Res. 125, 451–472. 10.1111/j.1365-2567.2007.02591.x 17496368

[B25] Steffes M. W. Schmidt D. Mccrery R. Basgen J. M. (2001). Glomerular cell number in normal subjects and in type 1 diabetic patients. Kidney Int. 59, 2104–2113. 10.1046/j.1523-1755.2001.00725.x 11380812

[B26] Tesch G. H. Allen T. J. (2007). Rodent models of streptozotocin-induced diabetic nephropathy. Nephrology 12, 261–266. 10.1111/j.1440-1797.2007.00796.x 17498121

[B27] Toffoli B. Zennaro C. Winkler C. Giordano Attianese G. Bernardi S. Carraro M. (2018). Hemicentin 1 influences podocyte dynamic changes in glomerular diseases. Am. J. Physiol. Ren. Physiol. 314, F1154–F1165. 10.1152/ajprenal.00198.2017 29488390

[B28] Unnikrishnan R. Pradeepa R. Joshi S. R. Mohan V. (2017). Type 2 diabetes: demystifying the global epidemic. Diabetes 66, 1432–1442. 10.2337/db16-0766 28533294

[B29] Weibel E. R. (1981). Practical methods for biological morphometry. J. Microsc. 121, 131–132. 10.1111/j.1365-2818.1981.tb01205.x.

[B30] Wu Y. Wang X. N. Chen F. F. Chen Y. N. Tian S. Q. (2013). Study on the effect of the active ingredients of Huidouba in the treatment of type 2 diabetes mellitus. J. Chin. Med. Mater. 36 (8), 1313–1316. CNKI:SUN:ZYCA.0.2013-08-034

[B31] Yang K. B. Pang Z. R. Bai Y. H. Lu B. N. Yu N. Han G. Y. (2020). Huidouba ameliorates kidney oxidative stress injury by down-regulating Nox4 expression in rats with diabetic nephropathy. Chin. J. Exp. Tradit. Med. Formulae 26 (18), 84–90. 10.13422/j.cnki.syfjx.20201839

[B32] Yang K. B. Pang Z. R. (2017). Research progress on treatment of diabetes with Tibetan medicine Huidouba. Chin. Tradit. Herb. Drugs 48, 1682–1686. 10.7501/j.issn.0253-2670.2017.08.031

[B33] Zhang L. Long J. Jiang W. Shi Y. He X. Zhou Z. (2016). Trends in chronic kidney disease in China. N. Engl. J. Med. 375, 905–906. 10.1056/NEJMc1602469 27579659

[B34] Zhang T. Gao Y. B. (2015). Clinical research on treatment of early diabetic nephropathy by replenishing Qi and nourishing Yin,Activating blood and dredging collaterals. World Chin. Med. 10, 1509–1511+1514. 10.3969/j.issn.1673-7202.2015.10.013

